# Electronic and Optical Properties of BP, InSe Monolayer and BP/InSe Heterojunction with Promising Photoelectronic Performance

**DOI:** 10.3390/ma15186214

**Published:** 2022-09-07

**Authors:** Xingyong Huang, Qilong Cao, Mingjie Wan, Hai-Zhi Song

**Affiliations:** 1 Computational Physics Key Laboratory of Sichuan Province, Faculty of Science, Yibin University, Yibin 644007, China; 2 Key Laboratory of Laser Device Technology, China North Industries Group Corporation Limited, Chengdu 610200, China; 3 Sichuan Province Engineering Technology Research Center of Powder Metallurgy, Chengdu University, Chengdu 610106, China; 4 Southwest Institute of Technical Physics, Chengdu 610041, China

**Keywords:** black phosphorus, indium selenide, van der Waals heterostructure, first-principle calculations

## Abstract

**Two-dimensional** (2D) materials provide a new strategy for developing photodetectors at the nanoscale. The electronic and optical properties of black phosphorus (BP), indium selenide (InSe) monolayer and BP/InSe heterojunction were investigated via first-principles calculations. The geometric characteristic shows that the BP, InSe monolayer and BP/InSe heterojunction have high structural symmetry, and the band gap values are 1.592, 2.139, and 1.136 eV, respectively. The results of band offset, band decomposed charge and electrostatic potential imply that the heterojunction structure can effectively inhibit the recombination of electron–-hole pairs, which is beneficial for carrier mobility of photoelectric devices. Moreover, the optical properties, including refractive index, reflectivity, electron energy loss, extinction coefficient, absorption coefficient and photon optical conductivity, show excellent performance. These findings reveal the optimistic application potential for future photoelectric devices. The results of the present study provide new insight into challenges related to the peculiar behavior of the aforementioned materials with applications.

## 1. Introduction

In 2004, the monolayer graphene was successfully prepared by K. S. Novoselov et al. [1]. Graphene exhibits a single atomic layer thickness, high carrier mobility (10^4^ cm^2^V^−1^s^−1^), thermal conductivity (4000 Wm^−1^K^−1^) and light transmittance (the absorbance of monolayer graphene is 2.7%) [2], And 2D materials have attracted much attention in the field of materials science [3,4,5,6,7].

However, the zero-bandgap characteristics of graphene lead to its limited application in the field of semiconductor materials, and alternative 2D materials have been actively explored. For example, 2D transition metal compounds (TMDs) (WSe_2_, MoS_2_, WS_2_, etc.) were successively prepared by mechanical exfoliation [8,9,10], which makes it possible to regulate the band gap of semiconductor materials. In addition, 2D TMDs exhibit special properties, such as excellent mechanical flexibility and thermal stability, and are widely used in electrochemical energy storage, and optical and electrical devices, which are expected to be prepared into molecular-level digital devices. The mobility of TMDs is low, which inspires researchers to explore new materials. In 2014, a field-effect transistor (FET) based on BP was prepared by Zhang. Y et al., which is a new semiconductor material after graphene and TMDs [11]. The band gap of BP can be tuned with thickness, and the whole spectrum from visible light to near-infrared region can be detected. In particular, the direct bandgap characteristics of BP enhance its direct coupling with light, resulting in tremendous potential in the field of photoelectric detection. Meanwhile, InSe, which has been described as the “golden section between silicon and graphene”, was studied [12]. The material, which is only a few atomic layers thick, shows superior electrical properties to silicon and has proved to be an excellent candidate for solar energy conversion [12,13].

Surprisingly, the ballistic avalanche phenomena was observed in nanoscale vertical InSe/BP heterostructures [14]. Moreover, the negative differential resistance was explored in van der Waals (vdW) BP/InSe FET [15], and FET based on the vdW BP/InSe heterojunction is a prospective way to improve tuning ability [16]. Theoretical research shows that the hole mobility exceeds 10^4^ cm^2^V^−1^s^−1^, and the electric field can be modulated in the InSe/BP heterojunction [17,18]. As far as we know, there are few kinds of research on comparing the electronic properties of the BP monolayer, InSe monolayer and BP/InSe heterojunction, and the systematic analysis of optical properties is especially rare. In this paper, the structural and electronic properties of BP, InSe monolayer and BP/InSe heterojunction were investigated, and the optical properties were systematically analyzed.

## 2. Methods

First-principles calculations were performed using the density functional theory (DFT) packaged in the Vienna ab initio simulation package (VASP) [19,20,21]. The generalized gradient approximation (GGA) with the Perdew–Burke–Ernzerhof (PBE) functional was chosen for electron exchange and correlation potentials [22]. The projector augmented wave (PAW) method was used to describe electron–ion interaction. The energy cutoff and convergence criteria of total energy and force were set to 400 eV, 10^−5^ eV and 0.01 eV/Å, respectively. The vacuum thickness is more than 15 Å in the *z*-direction. More accurate electronic and optical characteristics are obtained using Heyd–Scuseria–Ernzerhof (HSE06) hybrid functions [23].

## 3. Results and Discussions

**Figure 1:**
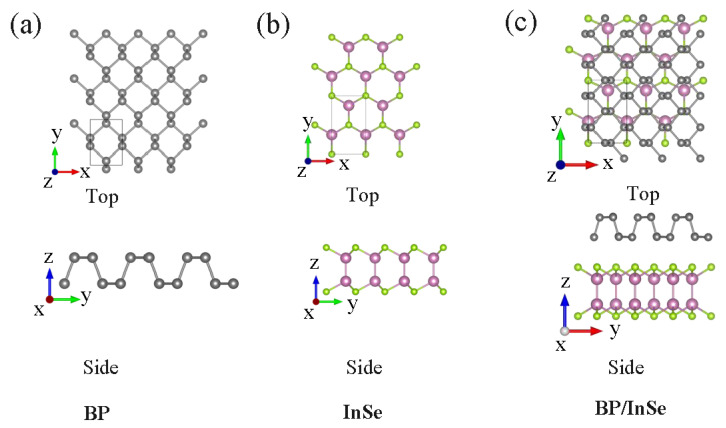
Top (upper panel) and side views (down panel) of the BP monolayer (**a**) and InSe monolayer (**b**), and BP/InSe heterostructure (**c**). The violet, green, and gray balls represent the In, Se, and P atoms, respectively.

The fully optimized geometric characteristic of the BP, InSe monolayer and BP/InSe heterojunction are shown in Figure 1. The BP monolayer (Figure 1a) shows the rectangle unit cell, and the lattice constants a and b are 3.298 and 4.623 Å, respectively, which is consistent with the previous experimental data (a = 3.32 Å; b = 4.58 Å) [24]. The primitive cell of the BP monolayer contains 4 P atoms. For the InSe monolayer (Figure 1b), it presents the hexagonal structure with a sequence of Se–In–In–Se, and each In (Se) atom forms three bonds with the neighboring Se (In) atoms. Moreover, the lattice parameter is 4.082 Å, which is consistent with the previous calculations (4.08 Å) [25]. Based on the minimization of the total energy and binding energy, BP/InSe heterojunction (Figure 1c) is constructed. As shown in Figure 1c, the optimized interlayer distance is 3.058 Å, which is a typical vdW heterojunction structure. Certainly, we considered the vertical heterostructures, and novel features lateral configuration have been discovered [26,27].

**Figure 2:**
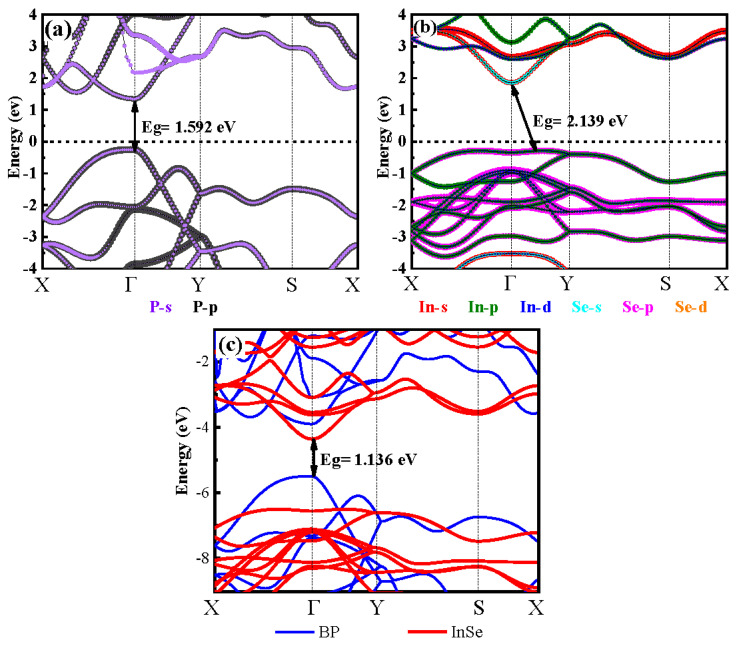
Calculated projected band structures for the InSe monolayer (**a**) and BP monolayer (**b**), and BP/InSe heterostructure (**c**) by the HSE06 method. The Fermi level is set at zero as the reference energy (**a**,**b**), and the vacuum level is taken as reference energy (**c**).

**Figure 3:**
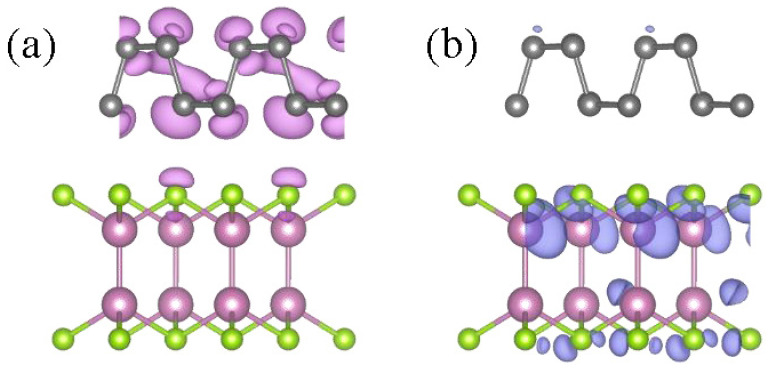
Isosurface plots of the band decomposed charge densities for the VBM (**a**) and the CBM (**b**). The isovalue is set to 0.004 e/Å^3^.

The HSE06-based projected band structure properties of the BP, InSe monolayer and BP/InSe heterojunction are shown in Figure 2. For the BP monolayer (Figure 2a), the bandgap value is 1.592 eV (agreement with the reported value (1.506 eV) [28], and the valence band maximum (VBM) and conduction band minimum (CBM) are both located the Γ point. In detail, the P-p orbit dominates the CBM and VBM. For the InSe monolayer (Figure 2b), it displays an indirect band gap (2.139 eV) semiconductor and the bandgap value is ’consistent with the reported value (2.121 eV) [29]. The VBM is located between the Γ and Y points and the CBM at the Γ point, and the energy difference is 57 meV between the direct and indirect band gaps. In addition, the valence band is mainly contributed by the Se-p and In-p states, while the conduction band is dominated by In-s states. Moreover, for the BP/InSe heterojunction, the detailed electronic properties can be obtained from Figure 2c. The obtained BP/InSe heterostructure is a direct semiconductor with a band gap of 1.136 eV, and the VBM and CBM are located at the Γ point. The CBM is mainly contributed by the InSe monolayer, and the VBM is mainly contributed by the BP monolayer. The VBM value of BP is greater than the VBM value of InSe and less than the CBM value of InSe, and the CBM value of BP is greater than the CBM value of InSe. The BP/InSe heterostructure is a type II heterojunction. The band offset of BP and InSe is ~1 eV for the VBM, while it is only ~0.4 eV for the CBM, reducing the recombination probability of electrons and holes, which is beneficial for carrier mobility of photoelectric devices. The meaningful discussion is the isosurface plots of the band decomposed charge densities for the CBM and the VBM (Figure 3). The VBM is mainly provided by the states from the BP monolayer, and the CBM is dominated by the states from the InSe monolayer. The holes and electrons are mainly localized in the InSe and BP regions, respectively. Spatial localization of the holes in CBM and the electrons in VBM is advantageous for the separation of electron–hole pairs in BP/InSe heterojunction, which is beneficial to improving the performance of photoelectric devices [30,31].

**Figure 4:**
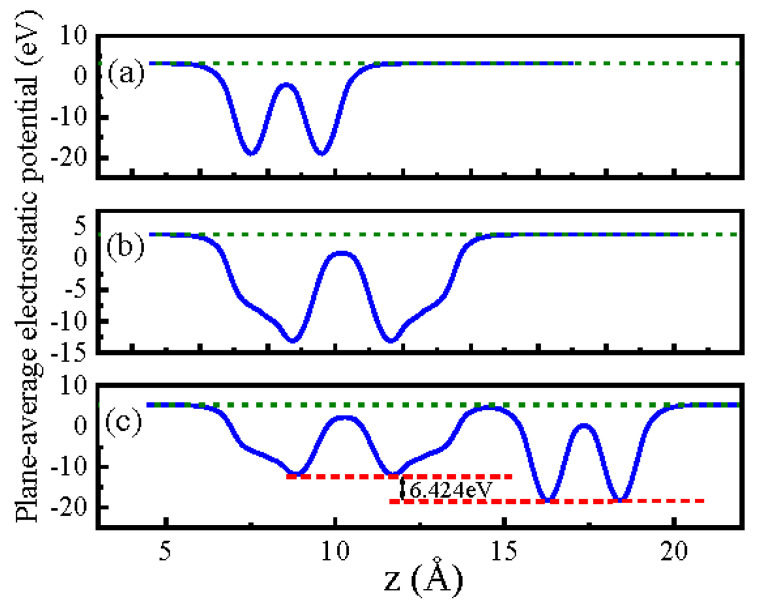
The plane-average electrostatic potential profile of the BP monolayer (**a**), InSe monolayer (**b**), and BP/InSe heterojunction (**c**) along the z-direction. The green dashed line denotes the vacuum level.

Furthermore, the plane-average electrostatic potential profile of the BP monolayer, InSe monolayer, and BP/InSe heterojunction along the z-direction are shown in Figure 4, respectively. Due to the structural symmetry of the BP monolayer (Figure 1a) and InSe monolayer (Figure 1b), the plane-average electrostatic potential profile of the BP monolayer (Figure 4a) and InSe monolayer (Figure 4b) also exhibits obvious symmetry. For the plane-average electrostatic potential profile of BP/InSe heterojunction (Figure 4c), there is a large potential drop (6.424 eV) between BP/InSe heterojunction interfaces, which implies that electrons are transferred from the BP monolayer to the InSe monolayer and an internal electric field is formed near the interface. Meanwhile, the large potential drops will achieve a low recombination rate for photoinduced electron–hole pairs [32], which is beneficial to improve the performance of photoelectric devices.

Using the photon frequency (ω)-dependent dielectric function (ε(ω)), optical properties (including extinction coefficient, refractive index, reflectivity, absorption coefficient, etc.) of a material can be derived [33]. ε(ω) can be expressed as $\varepsilon (\omega )=\varepsilon_{1}(\omega )\pm i\varepsilon_{2}(\omega )$, where $\varepsilon_{1}(\omega )$ and $\varepsilon_{2}(\omega )$ represent the real and imaginary parts of ε(ω), respectively. The ε(ω) can be obtained via Kramers–Kronig (KK) relation [34]. Generally, the $\varepsilon_{1}(\omega )$ is closely related to the polarization, while $\varepsilon_{2}(\omega )$ is closely related to the absorption properties [33]. $\varepsilon_{1}(\omega )$ and $\varepsilon_{2}(\omega )$ affect each other’s optical properties. For optical properties, in-plane and out-plane means the complex dielectric function for the light polarization in parallel (in-plane means *x*-direction or *y*-direction) and perpendicular to the plane (out-plane means *z*-direction), respectively. Since there is no significant difference in optical properties between *x* and *y* directions, we only study *x* (in-plane) and *z* (out-plane) directions here.

**Figure 5:**
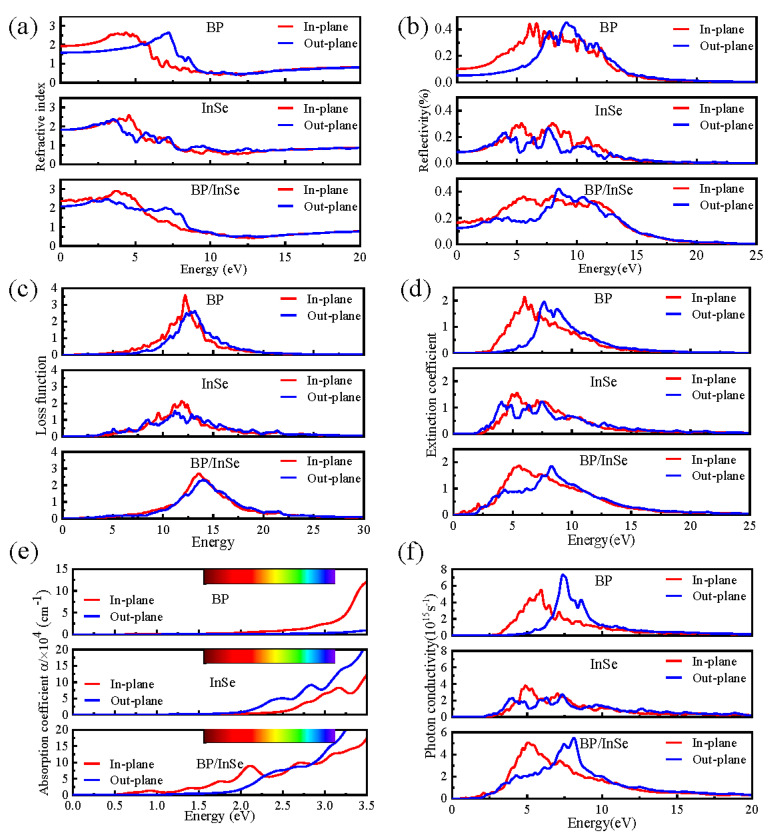
Optical properties of the BP monolayer, InSe monolayer and BP/InSe heterojunction. Graphs (**a**–**f**) represent refractive index, reflectivity, electron energy-loss spectroscopy, extinction coefficient, absorption coefficient, and optical conductivity, respectively.

Graphs displaying the dependence of refractive index on energy for BP, InSe monolayer and BP/InSe heterojunction are shown in Figure 5a. For all studied materials, the in-plane exhibits a higher refractive index than the out-plane in the low energy region (~<6 eV), and the opposite is true in the high energy region (~>6 eV). The maximum of the in-plane refractive index is located at ~5 eV, and the out-plane n is located at ~7.5 eV. Meanwhile, the in-plane refractive index of BP monolayer and out-plane refractive index of InSe monolayer are similar to the in-plane and out-plane refractive index profile curves of BP/InSe heterojunction, respectively. This means that the in-plane refractive index of the BP monolayer has a significant influence on the in-plane refractive index of BP/InSe heterojunction, while the out-plane refractive index of the InSe monolayer has an obvious influence on the out-plane refractive index of BP/InSe heterojunction; this phenomenon may be attributed to the heterojunction structure affecting the volume of the system [35].

The calculated reflectivity is shown in Figure 5b. For all studied materials, the reflectivity between 5 and 10 eV is almost between 30% and 40%. Overall, in-plane is higher than out-plane in the photon energy region (~<8 eV), mainly owing to the optical excitations in the plane being stronger than that out of the plane [34]. The BP/InSe heterojunction and BP monolayer are similar for the reflectivity curve profile, and BP/InSe heterojunction and InSe monolayer are similar for the reflectivity values, mainly owing to the presence of a possible electric field in the heterojunction [34].

The electron energy-loss spectroscopy (EELS) is represented in Figure 5c. The value of the EELS is related to the feature coupled with plasma oscillation [34]. The maximum electron energy loss for BP, InSe monolayer, and BP/InSe heterojunction is ~13 eV, and the in-plane is higher than the out-plane. Moreover, the maximum electron energy loss of BP/InSe heterojunction is higher than the InSe monolayer and lower than the BP monolayer, which is dominated by the imaginary part $\varepsilon_{2}(\omega )$ of the dielectric function [35].

Figure 5d shows the extinction coefficient. The in-plane extinction coefficient maxima are all at ~5 eV, while the out-plane extinction coefficient maxima are all at ~8 eV. The maximum position of the extinction coefficient of BP/InSe heterojunction is close to that of the InSe monolayer. Due to the profile characteristics of the imaginary part $\varepsilon_{2}(\omega )$ of the dielectric function, the curve profile is similar to that of the BP monolayer [36].

Figure 5e shows that the absorption coefficient increases with increasing photon energy, and the absorption coefficient value can reach 10^4^ cm^−1^ in the photon energy region (~>3 eV). The absorption performance of the BP monolayer is unsatisfactory, covering the ultraviolet region, which is ~5% of the solar spectrum. Therefore, the visible and infrared regions of the spectrum cannot be absorbed efficiently by the BP monolayer. The InSe monolayer displays a slightly better absorption effect on blue light than the BP monolayer. In contrast, the BP/InSe heterojunction possessed excellent absorption performance with a suitable bandgap to achieve absorption of the visible region. It is evident that the imaginary component of the dielectric function almost interconnected with the absorption [36]. The in-plane absorption coefficient has a clear peak at ~2 eV and the value reaches as much as 10^5^ cm^−1^. The contrast analysis shows that the in-plane absorption coefficient of the BP/InSe heterojunction is similar to that of organic perovskite materials (10^4^–10^5^ cm^−1^) [37] and higher than that of many other 2D materials in visible light [21]. As such, the construction of the BP/InSe heterojunction is willing to improve the photoelectric conversion of solar energy. Certainly, excitons should be considered if the absorption properties are thoroughly explored [38,39].

The photoconductance effect is an important index of the photoconductance radiation detector, and the real part of the optical conductivity is presented (Figure 5f) as an optical property. All the photoconductance starts at an energy of ~2.5 eV, and the maximum value in-plane (out-plane) is at ~5 eV (~8 eV). The in-plane conductivity of the low-energy region (<~7 eV) is higher than that of the out-plane region, while that of the high-energy region (>~7 eV) is the opposite. The overall conductivity performance of the BP/InSe heterojunction is between BP monolayer and the InSe monolayer. Since the material generates conductive free carriers when absorbing energy, the conductivity spectrum has similar characteristics to the absorption spectra [35].

## 4. Conclusions

The structural, electronic and optical properties of the BP, InSe monolayer, and BP/InSe heterojunction are investigated systematically. Both BP and InSe monolayer are highly symmetrical. Based on the HSE06 method, the band gaps of the BP, InSe monolayer and BP/InSe heterojunction are calculated to be 1.592, 2.139 and 1.136 eV, respectively. For BP/InSe heterojunction, the electrons and holes are localized in the VBM and CBM, respectively. The plane-average electrostatic potential profile of BP/InSe heterojunction presents a large potential drop (6.424 eV) between BP/InSe heterojunction interfaces. The refractive index, reflectivity, electron energy loss, extinction coefficient, absorption coefficient, and photon optical conductivity exhibit excellent optical properties. The research material has finite energy gaps and has promising potential in the field of 2D optoelectronic devices. We acquired an innovative technique for the precursive essences to analyze the considered material which is implemented in diverse engineering applications along with medical physics.

## Data Availability

Data available on request. The data presented in this study are available on request from the corresponding author.
